# Pushing the boundaries: an interview with Dae-Hyun Kim on terahertz devices

**DOI:** 10.1093/nsr/nwae013

**Published:** 2024-01-10

**Authors:** Defu Wang

**Affiliations:** Defu Wang is a Research Associate Professor at the Center for Carbon-based Electronics, School of Electronics, Peking University

## Abstract

*III–V compound semiconductors, such as InGaAs/InAlAs, exhibit exceptional carrier transport properties, establishing them as fundamental elements in terahertz (THz) applications crucial for the development of 6G networks. These materials present the potential for high-performance, energy-efficient THz devices. Furthermore, their compatibility with heterojunction integration, particularly in hetero-integration with silicon-germanium (SiGe) bipolar complementary metal–oxide–semiconductor (BiCMOS), paves the way for cutting-edge THz devices. This advantage highlights the crucial role of III–V semiconductors in driving THz and 6G technology, meeting the evolving demands of future wireless communication and sensing systems*.

*NSR conducted an interview with Dr. Dae-Hyun Kim, a semiconductor expert with a distinguished academic and professional background. Dr. Kim embarked on his career at Teledyne Scientific Company in 2008, later joining SEMATECH in 2012, where he played a crucial role in propelling semiconductor technology forward. In 2015, he assumed the position of an Associate Professor at Kyungpook National University. Dr. Kim's journey embodies his long commitment to the field, underscored by his remarkable contributions. In this interview, Dr. Kim shared his insights on fostering collaboration within the semiconductor field. He particularly emphasized on effective approaches for advancing research and innovation in semiconductor technology*.

## THz DEVICES: PIONEERING 6G AND BEYOND

***NSR*:** Why research THz devices for 6G, and why are they important for 6G technology?

***Kim*:** THz technology is emerging as a cornerstone in the evolution of 6G networks, primarily due to its capability to provide large contiguous frequency bands. This attribute is crucial in meeting the burgeoning demand for extremely high data transfer rates, potentially reaching the Tbit/s range. The transition from 5G to 6G is marked by the necessity to achieve significant improvements in key performance indicators (KPIs) such as peak data rates, user-experienced data rates and user plane latency. THz technology stands out as a pivotal solution in this transition, addressing the escalating performance requirements of 6G networks. The potential of THz technology extends beyond just communication; it encompasses applications in high-speed communications, imaging, sensing, non-destructive evaluation and various industrial applications. This technology is particularly promising for enabling ultra-fast data transfer rates and non-invasive imaging and sensing techniques. These capabilities are invaluable across various industries and scientific research domains, offering transformative solutions for numerous applications.

***NSR*:** What engineering strategies show potential for achieving high-frequency transistors for 6G and beyond, and what key engineering design principles are crucial for success in this area? Additionally, regarding industrial fabrication and the use of a 3-inch indium phosphide (InP) substrate in your work, can this

**Figure:**
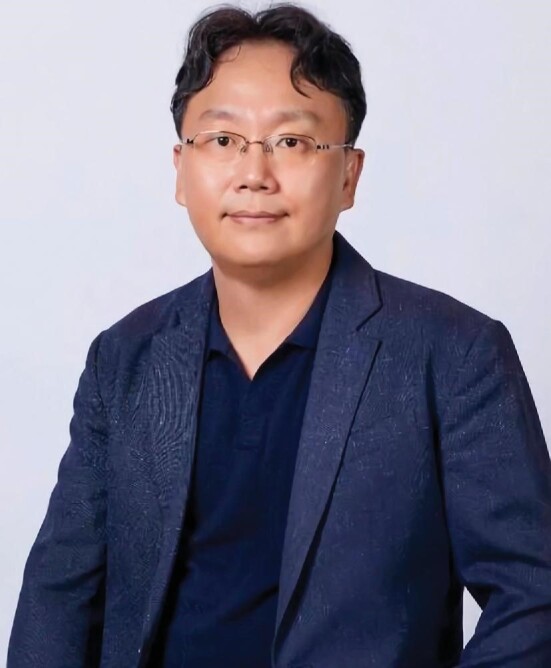
Dae-Hyun Kim, a pioneer in semiconductor research, achieved a historic milestone by fabricating III–V transistors with *f_t_* (transit frequency) and *f*_max_ (maximum oscillation frequency) exceeding 700 GHz (*Courtesy of Prof. Dae-Hyun Kim*).

substrate's capabilities be extended further, and do you see the possibility of making breakthroughs in this area?

Developing new nanofabrication techniques is essential for creating high-frequency and high-power transistors capable of supporting the demanding requirements of 6G networks and beyond.

—Dae-Hyun Kim

***Kim*:** One crucial approach involves the development of advanced radio frequency (RF) switches. These switches are vital because they offer low-energy consumption and high-speed functionality, which is particularly important for the growing number of battery-operated devices that will access the 6G network. Another exciting avenue is THz communications. This technology holds immense promise for 6G and beyond due to the enormous bandwidth available in the THz band. We believe that exploring this potential is key to meeting the expected peak data-rate demands of up to one terabit per second for 6G networks. Lastly, we must focus on nanofabrication methods. Developing new nanofabrication techniques is essential for creating high-frequency and high-power transistors capable of supporting the demanding requirements of 6G networks and beyond. In terms of design principles, efficiency is paramount.

We need to make our components, such as switches, more efficient. This is critical for achieving the higher speeds and power efficiency that 6G devices demand. Improved efficiency also has far-reaching implications, extending battery life and reducing latency, particularly in applications like autonomous vehicles and smart cities. Another fundamental principle is bandwidth exploration. We must thoroughly investigate the vast potential offered by the THz band's substantial bandwidth to meet the anticipated data-rate peaks in 6G networks. Additionally, advancements in nanofabrication techniques are pivotal. These advances are necessary to create transistors with high-frequency capabilities and substantial power output, which are essential for supporting the evolving needs of 6G networks and their subsequent iterations.

Now, in terms of industrial fabrication, the use of a 3-inch InP substrate is a crucial element. This substrate is integral to the development of high-frequency and high-power transistors that can effectively support 6G networks and their future iterations. Furthermore, we see the potential to enhance the capabilities of the 3-inch InP substrate through ongoing advancements in nanofabrication methods and materials research. This presents us with exciting opportunities for growth and innovation in this domain. To achieve breakthroughs in this area, we need to focus our research and development efforts on understanding and improving the performance of advanced semiconductor materials, such as gallium nitride and complex oxide materials. These materials hold the key to unlocking new possibilities in the field of semiconductor fabrication.

In conclusion, our journey to high-frequency transistors for 6G and beyond hinges on these engineering strategies and design principles. Moreover, the use of the 3-inch InP substrate in industrial fabrication represents a significant milestone with tremendous potential for future advancements. By addressing these aspects collectively, we are poised to shape the landscape of 6G technology and beyond.

## NEW APPLICATIONS AND CHALLENGES FOR III–V THz SEMICONDUCTOR DEVICES

***NSR*:** Which application opportunities do you find most promising concerning III–V THz semiconductor devices?

***Kim*:** THz band communication is not just an emerging technology; it is seen as a critical component for the future of telecommunications, particularly for 6G and beyond. The need for higher data-rate communication is ever-increasing, and the THz band is poised to meet this challenge. Researchers have already made significant strides in this domain, with experiments demonstrating data transmission speeds of up to 100 gigabits per second at THz frequencies. This rate is a ten-fold increase compared with the capabilities of current 5G technology.

The implications of achieving such high data rates are far-reaching. THz band communication can support a wide array of demanding applications. This includes extended reality (XR) and holographic teleportation, which require immense bandwidth and low latency. It also plays a crucial role in Industry 4.0 and the development of digital twins, where real-time data transfer and processing are critical. Additionally, applications in Cooperative, Connected, and Automated Mobility (CCAM), Non-Terrestrial Networks (NTNs) and Vehicleto-Infrastructure (V2I) communications will greatly benefit from the high data rate and low latency that THz technology can provide.

As we move forward, continuous research and development are essential to address these challenges and fully harness the potential of high-frequency semiconductor devices.

—Dae-Hyun Kim

***NSR*:** What practical applications do you envision for InGaAs-based high-electron-mobility transistors (HEMTs), given your expertise in compound semiconductors and III–V materials? In the realm of post-complementary metal oxide–semiconductor (CMOS) logic, where do you foresee these devices offering significant advantages?

***Kim*:** InGaAs-based HEMTs are at the forefront of advancing post-CMOS logic technologies. Their practical applications and advantages are multifaceted. They are seen as a potential successor to the existing CMOS technology, especially as we approach the limits of the CMOS roadmap.

**Figure 1.**
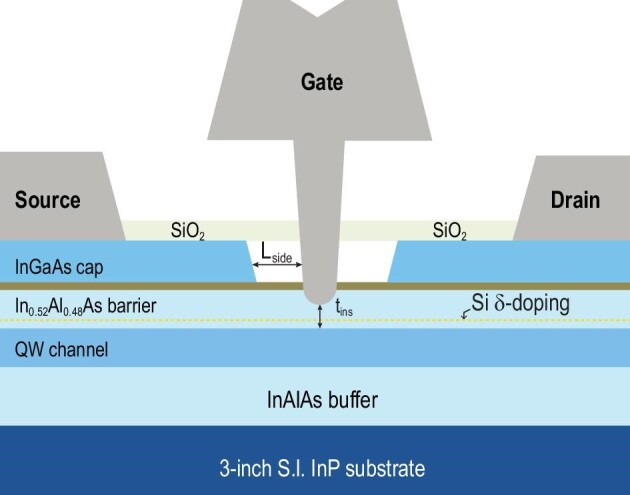
Schematic cross section of an In_0.8_Ga_0.2_As terahertz high-electron-mobility transistor with an *f_t_* of 0.75 THz and an *f*_max_ of 1.1 THz (Adapted from *IEEE T Electron Dev* 2023; **70**: 4).

Recently, InGaAs-based multi-bridge-channel field effect transistors (MBCFETs) (Fig. 1) have demonstrated remarkable transconductance values and high cut-off frequencies, offering notable advantages and practical applications in various areas. These devices are critical due to their high performance in terms of speed and efficiency, making them well suited for high-frequency applications that are essential for 6G technologies. They demonstrate remarkable potential in applications ranging from high-speed logic to RF applications and even non-invasive sensing, making them versatile in both industrial and scientific applications.

Moreover, the integration of these semiconductor devices into the existing CMOS technology platforms highlights their compatibility with current manufacturing processes. This compatibility is crucial for the seamless transition to advanced technologies without necessitating complete overhauls of existing fabrication infrastructures.

However, challenges remain, particularly in terms of scalability and integration with other components in the 6G architecture. As we move forward, continuous research and development are essential to address these challenges and fully harness the potential of high-frequency semiconductor devices.

## ADVANCING INITIATIVES AND PROSPECTIVE PATHWAYS

***NSR*:** Can the fundamental principles of THz electronics be extended to other applications? What are your insights into the future evolution of these technologies?

***Kim*:** The realm of THz electronics is on the cusp of extending its fundamental principles to a plethora of applications beyond its current scope. This technology, due to its unique attributes like the ability to penetrate most non-conducting materials, has garnered significant interest.

Moreover, THz solid-state devices are poised to bridge the gap between photonics and electronics, which could revolutionize information communication technology. This technology has already found applications in compact radar systems, enhancing the capabilities of radar and communication systems.

Looking ahead, a major focus in the evolution of THz technologies lies in developing high-power THz radiation. This challenge is substantial and transcends material or principle considerations. Additionally, photonic integrated circuits are expected to play a crucial role in the successful deployment of THz technologies. These circuits offer design and material flexibility, which can be leveraged to enhance the efficiency and effectiveness of THz applications.

The heterogeneous integration, particularly of InP with SiGe BiCMOS, opens up promising paths for innovation in high-frequency transistor research, crucial for 6G and beyond.

—Dae-Hyun Kim

***NSR*:** Do you see potential applications of related approaches in the development of heterojunctions with SiGe BiCMOS for 6G devices? Additionally, are there other promising opportunities in the realm of THz sensing and imaging that you find noteworthy?

***Kim*:** In the realm of semiconductor devices for 6G applications, substantial strides have been taken in high-frequency transistor research within SiGe BiCMOS and III–V technologies.

SiGe BiCMOS technologies have garnered attention for their potential to enhance the performance of silicon transistors. This advancement is crucial as it leverages the cost-effectiveness and scalability of established high-volume silicon manufacturing, particularly for 6G sub-THz applications. The synergistic combination of heterojunction bipolar transistors (HBTs) and CMOS technology offers the optimal mix of analog and digital performance. This is key for integrating RF functionalities into smart electronic chips, making them more efficient and versatile.

Concurrently, III–V technologies that are actively investigated include InP and gallium nitride (GaN) for their superior front-end performance at sub-THz frequencies. However, the challenge lies in addressing the cost and scalability issues associated with these materials. Advancements in both heterogeneous and monolithic integration with silicon are essential to overcome these hurdles.

Looking ahead, key avenues for future innovation include advancements in heterogeneous integration, power amplifier benchmarks and nanofabrication materials. The heterogeneous integration, particularly of InP with SiGe BiCMOS, opens up promising paths for innovation in high-frequency transistor research, crucial for 6G and beyond. This integration could be a game changer in developing more efficient and cost-effective high-frequency devices. Another critical area of innovation is in the development of power amplifiers, especially in the beyond 200 GHz frequency band. This is a key frontier in high-frequency transistor research, offering significant potential for advancements in 6G technologies. The role of nanofabrication methods cannot be overstated. These advancements are vital for creating transistors that can operate at high frequencies and high power, which are essential requirements for supporting 6G networks and beyond.

